# Role of neutrophil-lymphocyte-ratio in the mortality of males diagnosed with COVID-19

**Published:** 2020-06

**Authors:** Tahir Belice, Ismail Demir, Arif Yüksel

**Affiliations:** Department of Internal Diseases, Turkish University of Health Sciences, Izmir Bozyaka Training and Research Hospital, Izmir, Turkey

**Keywords:** COVID-19, Neutrophils, Lymphocytes

## Abstract

**Background and Objectives:**

With this study, for the first time among patients diagnosed with COVID-19, the neutrophil-lymphocyte-ratios of men and women were compared.

**Materials and Methods:**

The study was conducted with 80 patients and the data was gained retrospectively on the electronic documents of the hospital.

**Results:**

The neutrophil-lymphocyte-ratio was statistically significant and higher in the male than the women for all ages and geriatric patients (p<0.05).

**Conclusion:**

The higher neutrophil-lymphocyte-ratio in older males diagnosed with COVID-19 could be a causative reason for the higher mortality rates in men. We hope that these findings would be helpful for further studies.

## INTRODUCTION

COVID-19, which is the acronym of ‘’coronavirus disease 2019”, continues to emerge and represents a chaotic problem to the health system (1). Several viral epidemics have been recorded, such as SARS-CoV, H1N1 influenza, and MERS-CoV so far, but we have never reached to the point that is named as a pandemic that much easily and fast (2). COVID-19 is caused by a very contagious virus that is quickly spreading globally. First, in Wuhan, which is in China’s Hubei province, it was reported to the WHO Country Office as cases with unknown triggers lead to respiratory infections (3). But now it is almost in every part of the world living with most people and probably will continue in the people who survive through a process such as natural selection base on the type of angiotensin-converting enzyme receptor, age, or gender, etc. (4, 5). Almost in one-third of older adults aged 65 and over, infections are the leading causes of death, and not only mortality but also related morbidities increase beside (6). Infections trigger and exacerbate the geriatric syndromes concluding with functional dysfunction and reduced quality of life, and possibly on every occasion, they cause damage to organs to some extent (7). Because of the immunosenescence, older people are susceptible to infections, and the data about age-related changes in immune function are limited by an incomplete understanding of the complexities of the immune mechanism. Although aging does not affect the total number of neutrophils in the blood, the ability of phagocytosis has been shown to decrease in older people (8, 9). T lymphocyte is also another type of immune cell affected by the aging process, and it is known as a critical factor of the adaptive immune system, especially in limiting and clearing virus infections (10, 11). In a study, neutrophil-lymphocyte-ratio (NLR) was found to be superior to CURB-65 and an independent predictor for unfavorable outcomes (12). Patients diagnosed with COVID-19, especially in severe cases, have higher NLR and higher leukocytes counts with a lower number of lymphocytes (13). COVID-19 is more likely to occur in older men, and the reason why it is mostly mortal in men is an important question to answer (14, 15). With this study we reveal for the first time that the men have a higher NLR than the women who were charged in the hospital because of their clinical symptoms of COVID-19 and this study will give an idea for further studies that would decrease the mortality rates.

## MATERIALS AND METHODS

We saved the age, gender, neutrophil counts, and lymphocyte counts of participants from the Probel software of the hospital network, which enabled us to have the electronic documents of patients. All the patients who charged in the hospital after diagnosed with COVID-19 based on the clinical and radiological findings in the emergency unit were included in this study. On the date of 07.04.2020, the laboratory findings of the 80 patients in the pandemic wards of the hospital were saved in excel files. All statistical analyses of the frequency rates, percentages, proportions, means, correlations, and standard deviations were performed using SPSS. While in the continuous variables that were normally distributed, independent T-tests were used; for the continuous variables not normally distributed, we used the Mann-Whitney U for the comparison of means. Proportions for categorical variables were compared using the χ^2^ test. Two-sided P-value less than 0.05 was considered statistically significant.

All procedures performed in studies involving human participants were in accordance with the ethical standards of the institutional research committee (Turkish Turkish University of Health Sciences, Izmir Bozyaka Training and Research Hospital Izmir Bozyaka Education and Training Hospital) and with the 1964 Helsinki declaration and its later amendments or comparable ethical standards.

## RESULTS

In the study, a total of 80 patients diagnosed with COVID-19 were compared by age, gender, and NLR in SPSS. The gender distribution is as follows: 46 male (57,5%) and 34 women (42,5%). While the number of patients aged 65 and over is 32 (60%) with 12 (37,5%) female and 20 (62,5%) male, the number of patients under 65 years old is 48 (40%) with 22 (45,8%) female and 26 (54,2%) male. As patients age, the NLRs increased significantly, and also patients aged 65 and over have a higher NLR than patients aged under 65 (p<0.05). The NLR is statistically significant and more higher in the men than the women for all ages and geriatric people (p<0.05). But under 65 years old, we did not find any significant difference in NLR between sexes (p>0.05). The mean NLR is 5,70 +/− 0,9 in male and 3,38 +/− 0,8 in female across all ages. For patients aged 65 and over, mean NLR is 9,1 +/− 0,5 in men and 4,4 +/− 0,8 in women, whereas patients under 65 years old are found that mean NLR is 3,4 +/− 0,7 in male and 2,8 +/− 0,8. In other words, being a man and aged 65 and over are two risk factors for COVID-19.

## DISCUSSION

As we experienced until now that not only in SARS and MERS-CoV infection but also recently in COVID-19, there is a high proportion of pro-inflammatory cytokines in the serum of many patients, which could be the main factor on the mortality rates of those viruses (16, 17, 18). COVID-19 has not been understood yet how it can easily spread all over the world and cause the fatal outcomes as well as chaotic problems in nations. As a new type, but also highly contagious disease, it seems to be devastating with an unknown pathogenicity mostly for older men (19). Although both gender-disaggregated data and information show that the number of cases is almost equal to each other, both the mortality rates and the status of vulnerability to the COVID-19 are not similar for both sexes, and the exact mechanism is not clear behind the gender differences (20). Still, we know that men cases are more severe than female cases, and the men differ from females in COVID-19 mortality rates resulting in a 2.8% fatality rate for male patients versus 1.7% for female patients (21). Nearly half of COVID-19 patients are older, and men are more likely to be infected than women, and males account for a more significant percentage in the gender distribution of COVID-19 patients at around 60% (22, 23). In the presented study, we revealed that specifically, after 65 years old, the percentage of men (62,5%) in patients with COVID-19 was significantly higher than women (37,5%). We also found that NLR is statistically significant and higher in males than females both in all age groups and in the geriatric group (p<0.05). NLR differences according to gender may be as a result of potentially sex-based immunological or gendered differences (hormones, social roles, sex chromosomes, anatomical variations, etc.) (24, 25). We found that the mean NLRs of men and women aged 65 and over were 9,1 +/− 0,5 and 4,4 +/− 0,8, respectively, whereas in men and women under 65 years old, the mean NLRs were found as 3,4 +/− 0,7 and 2,8 +/− 0,8 respectively. The mean NLRs of men and women were 5,70 +/− 0,9 and 3,38 +/− 0,8 respectively across all ages. In a study with 5000 healthy participants, the mean NLRs across all ages for men and women were 1.59 ± 0.59, 1.62 ± 0.64, respectively, and the female had a higher mean NLR than the male in younger groups (26). But differences in the mean of NLRs in this study were not that much various through gender and ages compare to the present study that mean NLRs were higher in men across all ages than in women. With a recently published article, we are now able to understand that the gender differences could also be based on a proposed theory, namely “ porphyrin theory”. This theory acclaims that the orf1ab, ORF3a, and ORF10 could coordinately attack heme on the beta chain of hemoglobin in patients that are infected by Coronavirus and as we know that men have a higher level of hemoglobin than women which could be a negative feature of men against the COVID-19 (27). X chromosome and sex hormones play an essential role in innate and adaptive immunity, and their roles in the human body could be a protective factor for women against COVID-19 (28, 29). Various findings in NLRs of patients could be attributed to those parameters that we mentioned previously, and that is the first study that compares the mean of NLRs with gender in patients diagnosed with COVID-19. We hope that these findings would be helpful for further studies.
